# Robotic resection of a single adenoid cystic tumor liver metastasis using ICG fluorescence. A case report and literature review

**DOI:** 10.3389/fsurg.2023.1162639

**Published:** 2023-03-23

**Authors:** Alessio Pasquale, Laura Marinelli, Francesco Antonio Ciarleglio, Michela Campora, Nick Salimian, Giovanni Viel, Alberto Brolese

**Affiliations:** ^1^ Department of General Surgery and Hepato-Pancreato-Biliary (HPB) Unit, APSS, Azienda Provinciale per I Servizi Sanitari, Trento, Italy; ^2^ Anatomy and Pathology Department, APSS, Azienda Provinciale per I Servizi Sanitari, Trento, Italy

**Keywords:** liver robotic surgery, liver metastasis, adenoid cystic carcinoma, ICG fluorescence, case report

## Abstract

Adenoid cystic carcinoma (AdCC) is a rare tumor that typically develops in the salivary glands and less frequently in other sites of the head and neck region. Only a few cases of resected metachronous liver metastases have been reported. Minimally invasive surgery is currently the gold standard of care for liver resections; furthermore, the use of Indocyanine Green (ICG) is continuously increasing in surgical practice, especially in cases of primary liver tumors and colorectal liver metastases, due to its capacity to enhance liver nodules. We report the case of a 54-year-old male with a single liver metastasis of AdCC, located in SIII, who presented in our center 9 months after resection of a primary tumor of the laryngotracheal junction and adjuvant proton therapy. A 25-mg injection of ICG (0.3 mg/kg) was administered 48 h before surgery in order to highlight the tumor and perform an ICG-guided resection. The lesion was clearly visible during surgery, and, given its position and the proximity to the main lobar vessels of the left lobe, we opted for a left lateral sectionectomy. The outcome was unremarkable, with no major postoperative complications. The administration of ICG 48 h before surgery seems to be a valid tool even in cases of AdCC liver metastases, providing surgeons with better visualization of the lesion and improving the precision of the resection.

## Introduction

Adenoid cystic carcinoma (AdCC) is a rare tumor with an incidence of 3–4.5 cases per million, accounting for 1% of all head and neck malignancies (1). It typically develops in minor or major salivary glands, but it can also arise from other sites in the neck, including the larynx, trachea, and bronchi, although this is extremely rare (2, 3). It is considered a slow-growing tumor with perineural spread (4) and a high incidence of local recurrence. Regional lymph node metastases are rare (5, 6, 7), while distant metastases occur in 19%–24% of AdCC cases with a mean presentation time of 7.6 years, although delayed recurrences can manifest even after 12–15 years (8), typically have an hematogenous spread involving the lungs, bones, brain, and liver (4, 9, 10)^,^. The disease has an unfavorable long term prognosis with survival rate at 5–10- 20 years around 68%-52%-28% (11).

Few studies are reported on the treatment of single metastases from AdCC; the preferred treatment and the role of surgery are still a matter of debate, and there is no consensus on the extent to which surgery improves overall survival (2, 12).

We present a rare case of a single liver metastasis from a laryngeal AdCC that arose 9 months after excision of the primary tumor and was treated with robotic liver resection and preoperative ICG injection.

Historically, ICG has been used for the quantification of liver function through the ICG retention rate at 15 min (ICG R15) and the plasma disappearance rate (ICG-PDR), in recent years, it has been increasingly used intraoperatively in the identification of liver lesions and is often used as an intraoperative navigation tool in conjunction with ultrasound.

## Case Presentation

A 54-year-old male was referred to our department for a 5.2 cm liver metastasis from a previous AdCC of the laryngotracheal junction. In January 2022, the patient underwent an *en bloc* resection of the pharynx, larynx, cervical oesophagus, and the first eight tracheal rings; a thyroidectomy and lymphadenectomy; anterolateral thigh free flap (ALTFF) reconstruction; and a definitive tracheostomy. Histopathological examination revealed grading of pT4a, N3b, G2, predominant tubular and cribriform pattern, with vascular and perineural invasion, and disease-free surgical margins (R0); external proton beam therapy was administered after the treatment.

**Figure 1:**
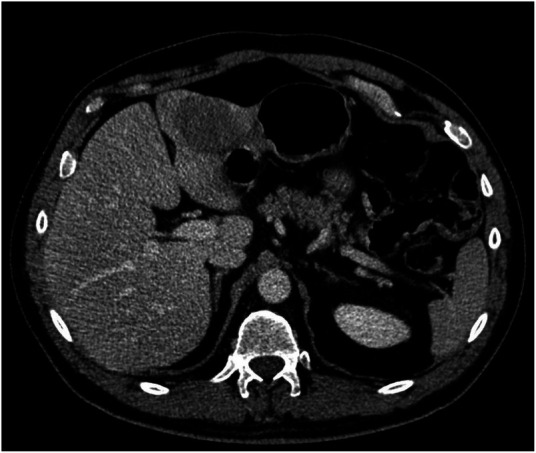
Abdominal CT scan showing a 5 cm lesion on segment 3.

A follow-up CT scan revealed a 4 cm liver lesion located between SII and SIII, suspected to be AdCC metastasis; a subsequent MRI confirmed the previous radiological findings (Figure 1). A percutaneous biopsy of the nodule was performed, and AdCC hepatic localization was diagnosed.

Further radiological evaluation with a total-body CT scan and an MRI of the neck revealed that the liver mass was increasing in size (5.2 cm) without evidence of local recurrence or extra-hepatic disease.

The case was discussed in a multidisciplinary team meeting. Given the patient's good performance status (ECOG 0), the presence of a single liver mass, and the favorable liver location in terms of liver resection, a robotic-assisted liver resection was scheduled. No major comorbidities were identified, nor were there any medical or surgical contraindications. The BMI and the ASA score were 30 and II, respectively.

According to the current literature forty-eight hours prior to surgery, 25 mg of ICG (0.3 mg/kg) were administered intravenously in an outpatient setting. The surgeon performed the procedure using a standard anterior robotic approach with the Da Vinci Xi Robotic Platform® (Intuitive Surgical; Sunnyvale, CA). The patient was placed in a supine position and a reverse Trendelenburg (30^°^). The docking was performed on the right side. Pneumoperitoneum was induced with an open technique in the left abdomen with a 12-mm AirSeal® port for the first assistant and a four-8 mm trocar transverse supraumbilical setup.

**Figure 2:**
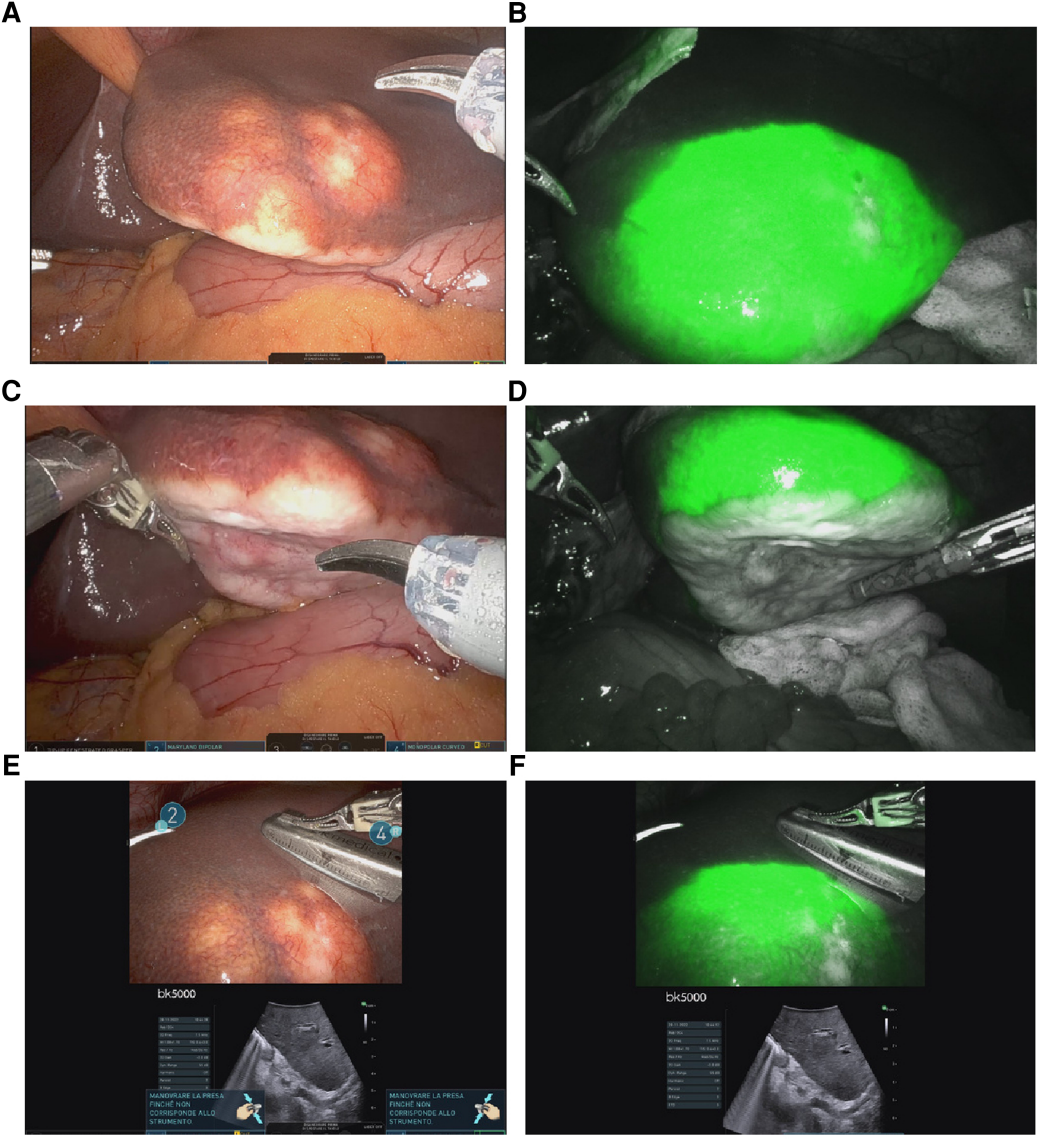
Intraoperative findings: (**A–C**) shows a single adCC liver metastasis located in the left lobe; (**B–D**): enhanced liver nodule using ICG; (**E–F**): combination of US and ICG fluorescence imaging in order to improve mass and margin visualization.

Explorative laparoscopy revealed no evidence of extrahepatic disease; an intraoperative US of the liver confirmed the sub-Glissonian lesion in SIII without additional hepatic localizations. When the camera was switched to the fluorescence setting, a perilesional tracer diffusion (rim pattern) was clearly seen around the lesion, no other nodules were found (Figure 2). Based on the intraoperative mass location, a decision was made to perform a left lateral sectionectomy. After hepatic pedicle preparation for a potential Pringle maneuver, the parenchymal transection was performed using the bipolar Maryland® forceps, scissors, and Vessel Sealer®; the pedicles for SII and SIII were divided between hemolocks, and the left hepatic vein was stapled with a 60 mm robotic stapler. No drain was placed in accordance with our standard procedure.

**Figure 3:**
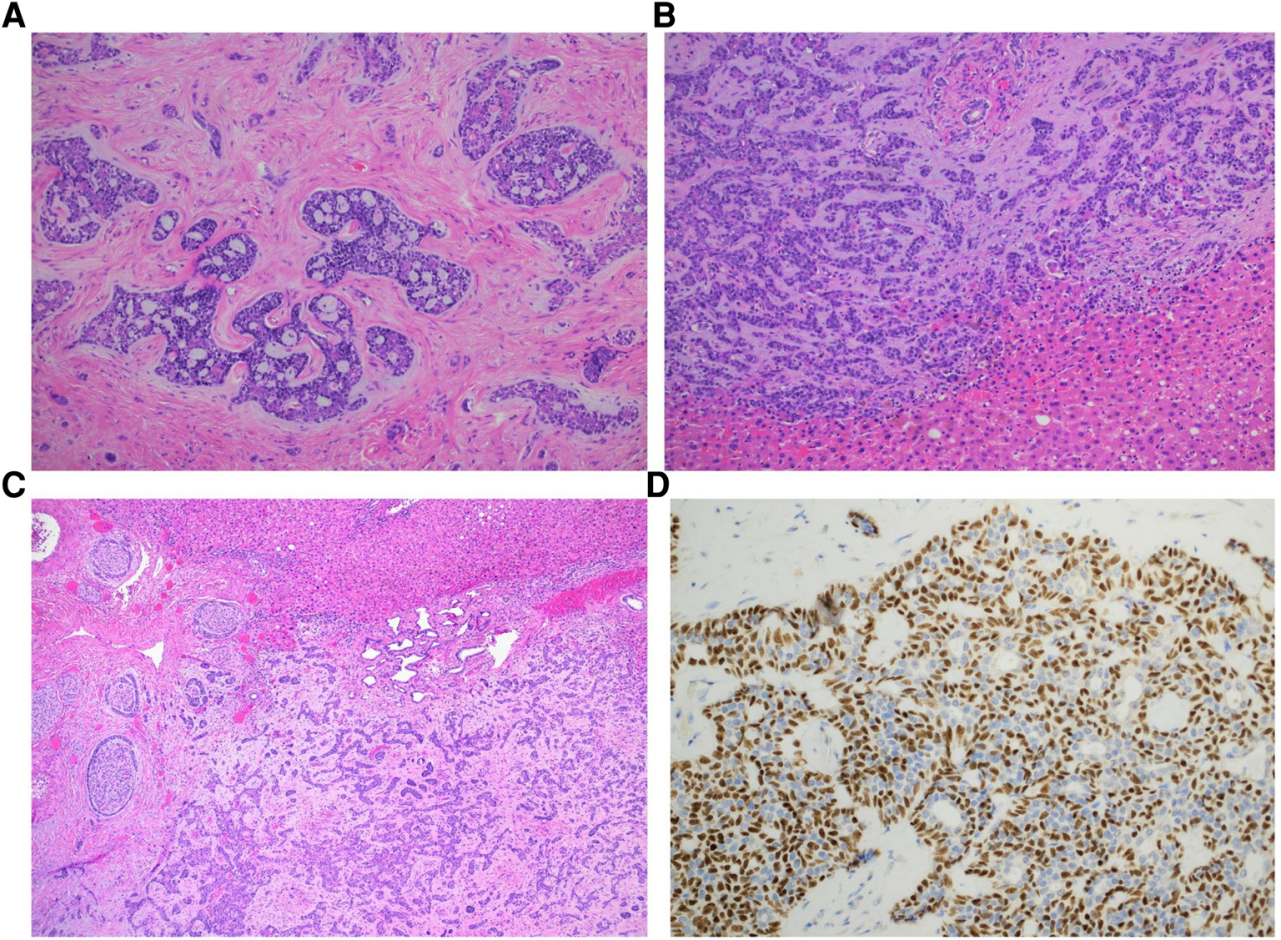
Histologic features of adenoid cystic carcinoma. Adenoid cystic carcinoma exhibiting predominantly cribriform (**A**) and tubular (**B**) growth patterns (hematoxylin and eosin stain, magnification 200x). Myxoid and hyalinized basement membrane material is noted in the cribriform area while tubular pattern contains simple tubules composed of inner ductal and outer myoepithelial cells. Perineural invasion is frequent (**C**, hematoxylin and eosin stain, magnification 400x). P63 immunostain highlights myoepithelial component and cribriform architecture (**D**, p63 magnification 400x).

The left lobe was placed in an endo-catch and subsequently extracted through a mini-Pfannenstiel. The duration of the operation was 192 min. No intraoperative complications occurred. Estimated blood loss was minimal (<100 ml); therefore, blood transfusions or intensive care were not required. The postoperative course was uneventful; the patient began oral feeding on postoperative day 1 and was discharged on postoperative day 3. The histological examination confirmed the presence of a neoplastic biphasic lesion with a prevalent tubular pattern, composed of ductal and myoepithelial cells respectively positive for CD117 and CK7/p63 at immunohistochemistry. Scattered areas with cribriform pattern, composed of predominantly myoepithelial cells with myxoid or hyalinized globules, were observed (Figure 3). Loss of biphasic differentiation, comedonecrosis, frequent mitoses and marked nuclear atypia, indicative of high grade transformation and associated with poor prognosis (13) were not identified. Perineural invasion and focal vascular invasion were present, and safe negative surgical margins (>2 cm) had been obtained. Final diagnoses of hepatic localization of AdCC was established.

The patient was disease-free at the three-month follow-up.

## Discussion

AdCC liver metastases are rare and typically progress slowly. Surgery seems to be a viable strategy for single lesions, also because there is no approved systemic therapy for metastatic AdCC (2). the median survival time after the appearance of distant metastases is 36 months (1 to 112 months) (11).

The identification of clinical, pathological and biomolecular prognostic factors is the first step in order to define the best treatment for each patient (11). There are some universally known clinical prognostic factors, such as presence and site of distant metastases, disease-free interval, lymphovascular invasion, grading, nodal metastasis, presence of extra-nodal extension, etc.; lately, many studies focused on some biomolecular factors that may help identify patients that must be treated more intensively and new therapeutic targets (11). Ferrarotto and colleagues. defined two different subtypes of AdCC through integrated clinical, genomic and proteomic analysis; the poor prognostic AdCC-I subtype seems to be associated with enrichment mutations in NOTCH1-a, SPEN, CREBPP and EP300, and appears to have an increased tendency to metastasize, particularly to the liver, while AdCC-II is associated with a better prognosis (14). Romani et al. demonstrated the prognostic relevance of multiple signalling pathways altered in AdCC, such as down-regulation of the myogenesis pathway and the enrichment of proliferation and cell-cycle related process (myc targets activation, increased expression of Ki-67 mRNA); they also discovered that an activation of IFN signalling in tumour cell has been linked to an unfavourable prognosis (15). Moreover, mutational pattern may change between primary and metastatic disease (11). Hence, a multidisciplinary approach is essential when planning the best strategy for these patients.

Metastases are generally metachronous, can arise even decades after the primary tumor removal (16), Coupland et al. reported a liver metastasis occurred more the 30 year (17), otherwise if synchronous they can represent the initial presentation of the disease (10, 18).

Some case reports and small series suggest that surgery could improve survival (3, 10, 12, 16); re-operation appears to be a feasible option in the case of a single hepatic recurrence, with an improvement in disease-free survival (19). Li XH et al. (10) recommended resections for isolated AdCC liver metastases due to the poor sensitivity to chemotherapy. More aggressive approaches have been proposed such as the treatment of unilobar multiple liver metastases with extensive hepatectomy after portal embolization, with an 18-month disease-free survival prior to a rapid reccurrence (20). Karatzas et al. described a combination of liver resection and radiofrequency ablation in cases of multifocal liver lesions that did not recur after one year (21), Ferrari et al. reported a case in which several recurrence were treated, at first lung metastasis and subsequent liver metastasis were resected. Subsequently simultaneous liver and kidney recurrence were subjected to surgery (a wedge resection and nephrectomy) and the patient was disease free after 2,5 years follow-up. In the report the authors argued that chemotherapy appears to be associated with a low response rate, and surgery represents the most curative treatment for distant metastasis (22) Table 1.

**Table 1:** Cases reported.

AUTHORS	Sex	Yrs	Primitive tumor	TNM	Radiotherapy	Other mts	Local recurrence	Liver mts	Time presentation	Chemotherapy	Treatment	Pathology	Follow-up
W. LIU ET AL, 2014	female	56	Larynx	No data	Yes	No	Yes	Single	4 yrs	No data	Resection	No data	Disease free at 6 years
L. XH ET AL, 2021	female	51	Sublingual gland	No data	No	No	No	Single Ø	Synchronous	No	Partial right hepatolobectomy	Tubiform and cribriform pattern	Disease free at 5 years
								41 mm					
I. ZEMNI ET AL, 2019	female	29	Parotid glands	T3N0M0	Yes	No	No	Single Ø	5yrs	Navelbine- CDDP	Wedge resection	Cribriform pattern	Disease free at 12 months
								210 X 90 mm					
K. NAKASHIMA ET AL, 2021	male	71	Submandibular gland	T2 N0 M0	No	No	No	Single Ø	5 yrs	No	Posterior sectionectomy	Tumor cells were positive for P63, Calponin, CK7, and CD117	Disease free at 6 months
								25 mm					
J. LI ET AL, 2020	female	69	Submandibular gland	No data	No	No	No	Single Ø	11 yrs	No	Left lateral section	Cribriform pattern	Disease free at 24 months
								56 × 70 mm					
A. COUPLAND, ET AL 2014	female	52	Submandibular gland	No data	Yes	No	Yes	Single Ø	>30 yrs	No	Wedge resection	Tubular and cribriform pattern	Disease free at 6 months
								25 mm					
G. SPOLVERATO ET AL, 2015	female	59	Submandibular gland	T3N2bM1	No	No	No	Single Ø	Synchronous	No	Extended left hepatectomy	Cribriform pattern	Disease free at 5 months
								45 mm					
V. SCUDERI ET AL 2011	female	30	Parotid gland	No data	Yes after local recurrence	Lung	Yes	Single Ø	11 yrs	Cisplatin, Doxorubicin, cyclophospamide	Left lateral section	Cribriform pattern	Recurrence after 21 months then redo surgery at 1 year follow-up: liver disease free with a stable lung disease
								120 × 80 mm					
G. BALDUCCI, ET AL, 2011	male	55	Submandibular gland	T2N2bMx	Yes	No	No	4 lesions	1 yrs	Cisplatin, Epirubicin	PVE + right extended hepatectomy	Ductal-like pattern	Recurrence at 24 months
A. KARATZAS ET AL, 2011	female	51	Submandibular gland	No data	No	No	No	8 lesions	Synchronous	Cisplatin, Doxorubicin, Epirubicin	TACE + extended right hepatectomy + RF	No data	Single recurrence after 3-months treated by RF, disease free after 1 year follow-up
C FERRARI ET AL, 2021	female	58	Submandibular gland	T3N2bM0	Yes	Kidney	No	Single Ø	>10 yrs	Cisplatin, Pharmorubicin	Wedge resection + right nephrectomy	No data	Disease free at 30 months
								10 mm					
						Lung		2° liver recurrence					

The use of ICG for the identification of primary and secondary liver tumors is a recent novelty and has been increasingly applied in recent years. ICG is completely eliminated from the livers of healthy subjects within 72 h. ICG may persist longer after IV administration in or around the liver lesions, with different stain pattern; in the hepatocellular carcinoma it's retained with typical central pattern (23), while in colorectal cancer metastases with a characteristic perilesional distribution (24) as described by Ishizawa et al. In the first case the distribution pattern is typically due to the tracer's accumulation in the cancer cells and their impaired secretion in the bile; in the second scenario the ICG is retained in the hepatocytes that have been compressed by the metastasis showing a typical rim pattern; the sensitivity of ICG in identifying liver metastases varies from 69% to 100% and is limited to lesions no deeper than 8 mm (25).

Currently, there is no unanimous consensus on the protocols to be used in the administration of indocyanine green (ICG) during liver surgery; typically, ICG is infused at a dose of 0.5 mg/kg from 7 days to 24 h before surgery (26).

The usefulness of ICG is not only linked to the identification of the lesion; in the context of minimally invasive resections, the negative or positive staining allows precise anatomical resections and it could reduce the recurrence rate (27). If administered intraoperatively, it is also considered useful for identifying a biliary leak and quality of the residual parenchyma; during cholecystectomy it has been also used for extrahepatic biliary anatomy identification.

To our knowledge, no other cases of hepatic resection for metastasis from AdCC of the laryngotracheal junction have been reported in the literature. Furthermore, preoperative ICG combined with intraoperative ultrasonography to improve mass and margin visualization can be considered a novel strategy for intraoperative liver mapping and treatment of this type of disease and could be considered an ideal navigation tool for hepatic resection for liver metastasis. Nowadays this combination strategy is not yet worldwide available and well established.

The main limitation of this case is that the exceptional rarity of the disease makes it impossible to make comparisons with previously documented cases published in the literature. Moreover, the actual short-term follow-up precludes the establishment of the true benefits for the patient in terms of disease-free survival and overall survival. In addition, the genetic characterization of the specimen that could suggest a more accurate prognosis or therapy is still unknow.

## Conclusion

Preoperative intravenous administration of ICG is currently standard practice for detection of superficial liver metastasis in our center. Furthermore, the combination of minimally invasive surgery, US, and preoperative ICG can be considered a valid option in select patients with metastatic nodules for real-time navigation during hepatic resections. The combination of preoperative IV ICG and intraoperative US appears to be an effective and useful strategy for liver surgeons in order to improve surgical strategies and outcomes.

Beyond the surgical aspects, it is important to emphasize the potential role of a precise molecular characterization that in the future could suggests the most appropriate and targeted treatment for each patient. Moreover, due to the natural history of the disease, a long-term follow-up is desirable.

## Permission to reuse and copyright

All claims expressed in this article are solely those of the authors and do not necessarily represent those of their affiliated organizations or those of the publisher, editors, or reviewers. Any product that may be evaluated in this article or claim that may be made by its manufacturer is not guaranteed or endorsed by the publisher.

This is an open-access article distributed under the terms of the Creative Commons Attribution License (CC BY). The use, distribution, or reproduction in other forums is permitted, provided the original authors and the copyright owners are credited and that the original publication in this journal is cited, in accordance with accepted academic practice. No use, distribution, or reproduction that does not comply with these terms is permitted.

## Data Availability

The original contributions presented in the study are included in the article/Supplementary Material, further inquiries can be directed to the corresponding author/s.

## References

[1] Coca-pelaz A Rodrigo JP Bradley PJ Vander V Rinaldo A Haigentz M Adenoid cystic carcinoma of the head and neck–An update. Adenoid Cyst Carcinoma Head Neck. (2015) 51:652–61. 10.1016/j.oraloncology.2015.04.005 25943783

[2] Lee RH Wai KC Chan JW Ha PK Kang H. Approaches to the management of metastatic adenoid cystic carcinoma. Cancers (Basel). (2022) 14(22):1–22. 10.3390/cancers14225698 PMC9688467 36428790

[3] Liu WEI Chen X. Adenoid cystic carcinoma of the larynx: a report of six cases with review of the literature. Acta Otolaryngol. (2015) 135(5):489–93. 10.3109/00016489.2014.990583 25743246

[4] Binesh F Akhavan A Masumi O Mirvakili A Behniafard N. Clinicopathological review and survival characteristics of adenoid cystic carcinoma. Indian J Otolaryngol Head Neck Surg. (2014) 67(1):62–6. 10.1007/s12070-014-0755-x 25621256 PMC4298605

[5] Ferlito A. Cervical lymph node metastasis in adenoid cystic carcinoma of the sinonasal tract, nasopharynx, lacrimal glands and external auditory canal: a collective international review. J Laryngol Otol. (2016) 130(12):1093–7. 10.1017/S0022215116009373 27839526 PMC5535774

[6] Amit M Na S Sharma K Ramer N Ramer I Eckardt M Elective neck dissection in patients with head and neck adenoid cystic carcinoma : an international collaborative study. Ann Surg Oncol. (2015) 22:1353–9. 10.1245/s10434-014-4106-7 25249259

[7] Chang C Hsieh M Chen M Chou M. Adenoid cystic carcinoma of head and neck : a retrospective clinical analysis of a single institution. Auris Nasus Larynx. (2018) 45(4):831–7. 10.1016/j.anl.2017.10.009 29653784

[8] van Weert S Reinhard R Bloemena E Buter J Witte BI Vergeer MR Differences in patterns of survival in metastatic adenoid cystic carcinoma of the head and neck. Head Neck. (2017) 39(3):456–63. 10.1002/hed.24613 27775851

[9] Khan AJ Digiovanna MP Ross DA Sasaki CT Carter D Son YH Adenoid cystic carcinoma: a retrospective clinical review. Int J Cancer. (2001) 96(3):149–58. 10.1002/ijc.1013 11410883

[10] Li XH Zhang YT Feng H. Liver metastasis as the initial clinical manifestation of sublingual gland adenoid cystic carcinoma: a case report. World J Clin Cases. (2021) 9(19):5238–44. 10.12998/wjcc.v9.i19.5238 34307573 PMC8283605

[11] Lorini L Ardighieri L Bozzola A Romani C Bignotti E Buglione M Prognosis and management of recurrent and/or metastatic head and neck adenoid cystic carcinoma. Oral Oncol. (2021) 115(January):105213. 10.1016/j.oraloncology.2021.105213 33578204

[12] Zemni I Tounsi N Bouraoui I Slimene M Sahraoui G Ayadi MA A single liver metastasis from adenoid cystic carcinoma of the parotid gland: case report. J Investig Med High Impact Case Rep. (2019) 7:4–7. 10.1016/j.oraloncology.2021.105213 PMC6764036 31556756

[13] Zhu Y Zhu X Xue X Zhang Y Hu C Liu W Exploration of high-grade transformation and postoperative radiotherapy on prognostic analysis for primary adenoid cystic carcinoma of the head and neck. Front Oncol. (2021) 11(April):1–11. 10.3389/fonc.2021.647172 PMC8063729 33898317

[14] Ferrarotto R Mitani Y McGrail DJ Li K Karpinets TV Bell D Proteogenomic analysis of salivary adenoid cystic carcinomas defines molecular subtypes and identifies therapeutic targets. Clin Cancer Res. (2021) 27(3):852–64. 10.1158/1078-0432.CCR-20-1192 33172898 PMC7854509

[15] Romani C Lorini L Bozzola A Bignotti E Tomasoni M Ardighieri L Functional profiles of curatively treated adenoid cystic carcinoma unveil prognostic features and potentially targetable pathways. Sci Rep. (2023) 13(1):1–9. 10.1038/s41598-023-28901-9 36720951 PMC9889376

[16] Nakashima K Misawa T Kumagai Y Kitamura H Fujioka S Yanaga K. Resection of liver metastasis from submandibular gland carcinoma five years after the primary operation: a case. Ann Med Surg. (2021) 62(January):373–6. 10.1016/j.amsu.2021.01.021 PMC7848741 33552497

[17] Coupland A Sewpaul A Darne A White S. Adenoid cystic carcinoma of the submandibular gland, locoregional recurrence, and a solitary liver metastasis more than 30 years since primary diagnosis. Case Rep Surg. (2014) 2014(June 2013):1–4. 10.1155/2014/581823 PMC4226169 25400973

[18] Spolverato G Fite J Bishop J Argani P author TMP. Liver metastasis as the initial presentation of adenoid cystic carcinoma title. Dig Dis Sci. (2014) 59(8):2004–6. 10.1007/s10620-014-3078-6 PMC4352306 24691627

[19] Scuderi V Ceriello A Romano M Migliaccio C Marsilia GM Calise F. Recurrent adenoid cystic carcinoma in the liver: a repeated laparoscopic surgical approach. Updates Surg. (2011) 63(4):301–6. 10.1007/s13304-011-0075-6 21647796

[20] Surgery G. Case report an unusual case of exclusive liver metastases from adenoid cystic carcinoma of the submandibular gland : a role for surgery ? Rep Case. (2011) 41(4):596–9. 10.1007/s00595-010-4318-9 21431502

[21] Reports C Karatzas A Katsanos K Maroulis I Kalogeropoulou C. Multi-modality curative treatment of salivary gland cancer liver metastases with drug-eluting bead chemoembolization, radiofrequency ablation, and surgical resection : a case report. J Med Case Rep. (2011) 5(1):416. Available at: http://www.jmedicalcasereports.com/content/5/1/416. 10.1186/1752-1947-5-416 21867491 PMC3170637

[22] Ferrari C Franceschi A Percivale A Griseri G. Surgical treatment of liver and kidney metastasis from an adenoid cystic carcinoma of the submandibular gland: case report. Tumori. (2021) 107(6):NP87–90. 10.1177/03008916211021664 34097534

[23] Takemura N Ito K Inagaki F Mihara F Kokudo N. Added value of indocyanine green fluorescence imaging in liver surgery. Hepatobiliary Pancreat Dis Int. (2022) 21(4):310–7. Available at: https://www.sciencedirect.com/science/article/pii/S1499387221002320. 10.1016/j.hbpd.2021.12.007 34953679

[24] Ishizawa T Fukushima N Shibahara J Masuda K Tamura S Aoki T Real-time identification of liver cancers by using indocyanine green fluorescent imaging. Cancer. (2009) 115(11):2491–504. 10.1002/cncr.24291 19326450

[25] Liberale G Bourgeois P Larsimont D Moreau M Donckier V Ishizawa T. Indocyanine green fluorescence-guided surgery after IV injection in metastatic colorectal cancer: a systematic review. Eur J Surg Oncol. (2017) 43(9):1656–67. 10.1016/j.ejso.2017.04.015 28579357

[26] Potharazu AV Gangemi A. Indocyanine green (ICG) fluorescence in robotic hepatobiliary surgery: a systematic review. Int J Med Robot. (2023) 19(1):e2485. 10.1002/rcs.2485 36417426 PMC10078519

[27] Felli E Ishizawa T Cherkaoui Z Diana M Tripon S Baumert TF Laparoscopic anatomical liver resection for malignancies using positive or negative staining technique with intraoperative indocyanine green-fluorescence imaging. HPB (Oxford). (2021) 23(11):1647–55. 10.1016/j.hpb.2021.05.006 34289953

